# Predicting stillbirth in a low resource setting

**DOI:** 10.1186/s12884-016-1061-2

**Published:** 2016-09-20

**Authors:** Gbenga A. Kayode, Diederick E. Grobbee, Mary Amoakoh-Coleman, Ibrahim Taiwo Adeleke, Evelyn Ansah, Joris A. H. de Groot, Kerstin Klipstein-Grobusch

**Affiliations:** 1 Julius Global Health, Julius Center for Health Sciences and Primary Care|University Medical Centre Utrecht, P.O. Box 85500, 3508 GA Utrecht, The Netherlands; 2 Department of Health Information, Federal Medical Centre Bida, Bida, Nigeria; 3 Ghana Health Service, Greater Accra Region Accra, Ghana; 4 Division of Epidemiology and Biostatistics, School of Public Health, Faculty of Health Science, University of Witwatersrand, Johannesburg, South Africa; 5 Global Geo and Health Data Center, Utrecht University, Utrecht, Netherlands

**Keywords:** Predicting, Stillbirth, Low-resource setting

## Abstract

**Background:**

Stillbirth is a major contributor to perinatal mortality and it is particularly common in low- and middle-income countries, where annually about three million stillbirths occur in the third trimester. This study aims to develop a prediction model for early detection of pregnancies at high risk of stillbirth.

**Methods:**

This retrospective cohort study examined 6,573 pregnant women who delivered at Federal Medical Centre Bida, a tertiary level of healthcare in Nigeria from January 2010 to December 2013. Descriptive statistics were performed and missing data imputed. Multivariable logistic regression was applied to examine the associations between selected candidate predictors and stillbirth. Discrimination and calibration were used to assess the model’s performance. The prediction model was validated internally and over-optimism was corrected.

**Results:**

We developed a prediction model for stillbirth that comprised maternal comorbidity, place of residence, maternal occupation, parity, bleeding in pregnancy, and fetal presentation. As a secondary analysis, we extended the model by including fetal growth rate as a predictor, to examine how beneficial ultrasound parameters would be for the predictive performance of the model. After internal validation, both calibration and discriminative performance of both the basic and extended model were excellent (i.e. C-statistic basic model = 0.80 (95 % CI 0.78–0.83) and extended model = 0.82 (95 % CI 0.80–0.83)).

**Conclusion:**

We developed a simple but informative prediction model for early detection of pregnancies with a high risk of stillbirth for early intervention in a low resource setting. Future research should focus on external validation of the performance of this promising model.

**Electronic supplementary material:**

The online version of this article (doi:10.1186/s12884-016-1061-2) contains supplementary material, which is available to authorized users.

## Background

Stillbirth is a major but silent contributor to perinatal mortality [1], and about 3 million third-trimester stillbirths [2, 3] occur annually, mainly (98 %) in low- and middle-income countries (LMICs) [4]. Despite several calls for action to reduce the rate of stillbirth [1, 4–8], stillbirths are yet to be addressed in the Global Burden of Disease metrics [9, 10], and Sustainable Development Goals [11]. Given that neither vital registration nor national stillbirth registers are adequately provided in LMIC [2, 12], together with the frequent omission from records of stillbirths that occur after 22 and before 28 weeks of gestation [13], the stillbirth rate has been underestimated. Studies have examined the associations between stillbirths and clinical [14–19] and non-clinical characteristics [20–22] of pregnant women but the knowledge generated is yet to have any positive impact on intrauterine survival in LMIC [23]. This indicates limited application of research findings to clinical settings, notably in low-resource settings, due to the inability of healthcare providers to combine these multiple predictors of stillbirth accurately to identify pregnancies with a high risk of stillbirth for early interventions [5, 6].

Therefore, it is important to develop an easy-to-apply clinical decision making tool for early detection of pregnancies with a high risk of stillbirth as recommended by experts in maternal and child health [12]. To date, only few attempts have been made to develop a decision making tool for early detection of pregnancies with a high risk of stillbirth but these models cannot be applied to low-resource settings. For example a prediction model for both stillbirth and neonatal death was developed in the United Kingdom [24] and subsequently validated in the United Kingdom and the Netherlands [25, 26]. This model predicts a different outcome (stillbirth and neonatal death in very preterm babies) and availability of routine data to validate it would be a great challenge in low-resource settings. Likewise, the prediction model developed by Akolekar et al. [27] contains some parameters such as Maternal Serum Pregnancy-Associated Plasma Protein-A and Reversed A-Wave in Ductus Venosus, that are not routinely assessed in low resource settings [27]. In this study we aimed to develop a prediction model to be applied in the second trimester of a pregnancy to identify pregnancies at high risk of stillbirth using routine clinical and non-clinical profiles of pregnant women who received care at a tertiary hospital in a low resource setting.

## Methods

### Study population

A retrospective cohort of 6,573 pregnant women that delivered at Federal Medical Centre Bida, a tertiary hospital in Niger state, Nigeria, from January 2010 to December 2013 was utilized to develop a prediction model for stillbirth. Only those women who delivered at the hospital after 20 completed weeks of gestation and gave birth to babies with no life-threatening congenital malformation were recruited.

### Data collection

Paper-based health records of all the included patients were retrieved from the Department of Health Information, Federal Medical Center Bida. Information was collected on clinical and non-clinical profile of the participants by the use of data extraction form in an anonymous format. Information on data extraction forms was transmitted to an electronic database using double data entry.

### Outcome

The outcome of the study was stillbirth, defined as fetal death that occurred after 20 completed weeks of gestation.

### Candidate predictors

For prediction modelling, the following candidate predictors were considered: maternal age, parity (number of previous pregnancies carried beyond viability i.e. up to 28 weeks gestational age), maternal education (woman who can read and write), maternal occupation, ethnicity, place of residence, previous fetal loss (number of previous pregnancy losses), bleeding in pregnancy (whether the woman had any complaint of vaginal bleeding during the index pregnancy), maternal height, number of previous caesarean sections, maternal weight, multiple gestation, sex, fetal presentation (part of the fetus closest the pelvic inlet, was categorized as cephalic, breech, and others), fetal growth rate (birth weight divided by gestational age at birth), and number of comorbid conditions. The following medical conditions, diagnosed by a physician were considered to generate a number of comorbid conditions: hypertension (defined as blood pressure of 140/90 mmHg and above) [28], pre-eclampsia (presence of hypertension and proteinuria) [28], diabetes (Diabetes is defined as Fasting Blood Sugar (FBS) > 7 mmol/L or 2-h Blood Sugar (RBS) > 11.1 mmol/L; Impaired Glucose tolerance is defined as Fasting Blood Sugar (FBS) 6.1–6.9 mmol/L or 2-h Blood Sugar (RBS) > 7.8–11 mmol/L) [29], sickle cell disease (presence of HbSS, HbSC or HbS β-thalassemia), renal disease (presence of clinical features, ultrasound findings, and elevated serum urea and creatinine), thyroid disease (presence of clinical manifestations and elevated serum free thyroxine and triiodothyroxine concentration) [29], syphilis (diagnosed using Venereal Disease Research Laboratory test) and pelvic inflammatory disease. All candidate predictors were selected based on availability, clinical experience and medical literature.

### Sample size calculation

We expected 2,000 deliveries per year and the incidence of stillbirth was assumed to be 4 % [30, 31]. Thus, 320 cases of stillbirths were expected to have occurred among 8,000 pregnant women who delivered at the hospital from 2010 to 2013. We planned to recruit all the 8,000 pregnant women who delivered at the hospital retrospectively. Given that at least 10 events to a potential predictor will be adequate to build a prediction model [32], we expected to have a sufficient number of events to build a robust prediction model.

### Data analysis

#### Descriptive statistics

Data were inspected and descriptive analyses performed using the complete dataset. Categorical data were described in terms of numbers and percentages while numerical data were expressed as median and interquartile range; the percentage of missing data in each potential predictor was determined.

#### Missing data

Multiple imputation technique using fully conditional specification was applied to impute missing data [33, 34].

#### Prognostic model

All potential predictors were entered into a multivariable logistic regression model and significant predictors were identified using stepwise backward selection with the Akaike Information Criterion (AIC) stopping rule. Predictors that were consistently retained in the model were selected and entered into a multivariable logistic regression. The best model was identified based on AIC and the results from each imputed dataset were pooled using Rubin’s rule [35]. Eventually, a prediction model for stillbirth was developed which we called the basic model. Subsequently, the basic model was extended with the variable fetal growth rate to become the extended model. The extended model was developed for those patients who had information on obstetric ultrasound, a procedure that is not routinely done in low-resource settings.

#### Performance of the model

The predictive performance of the final models was assessed by evaluating calibration and discrimination. Calibration determines the level of agreement between the observed events and model’s prediction and was presented by the calibration plot [36]. Discrimination examines how well the model can differentiate between participants with or without event and was expressed as C-statistic (which is equivalent to the area under the receiver operator curve) [37].

#### Internal validation

A bootstrap re-sampling technique was applied to the whole data to generate 200 testing datasets. The original models were re-fitted in the testing datasets and their shrinkage factors were estimated.

#### Model shrinkage

The shrinkage factor was used to adjust for over-optimism in each of the original models and the adjusted regression coefficients were calculated. The predictive performance of the final models was then re-assessed. All analyses were performed in R statistical software package [38].

## Results

### Patient characteristics

**Fig. 1:**
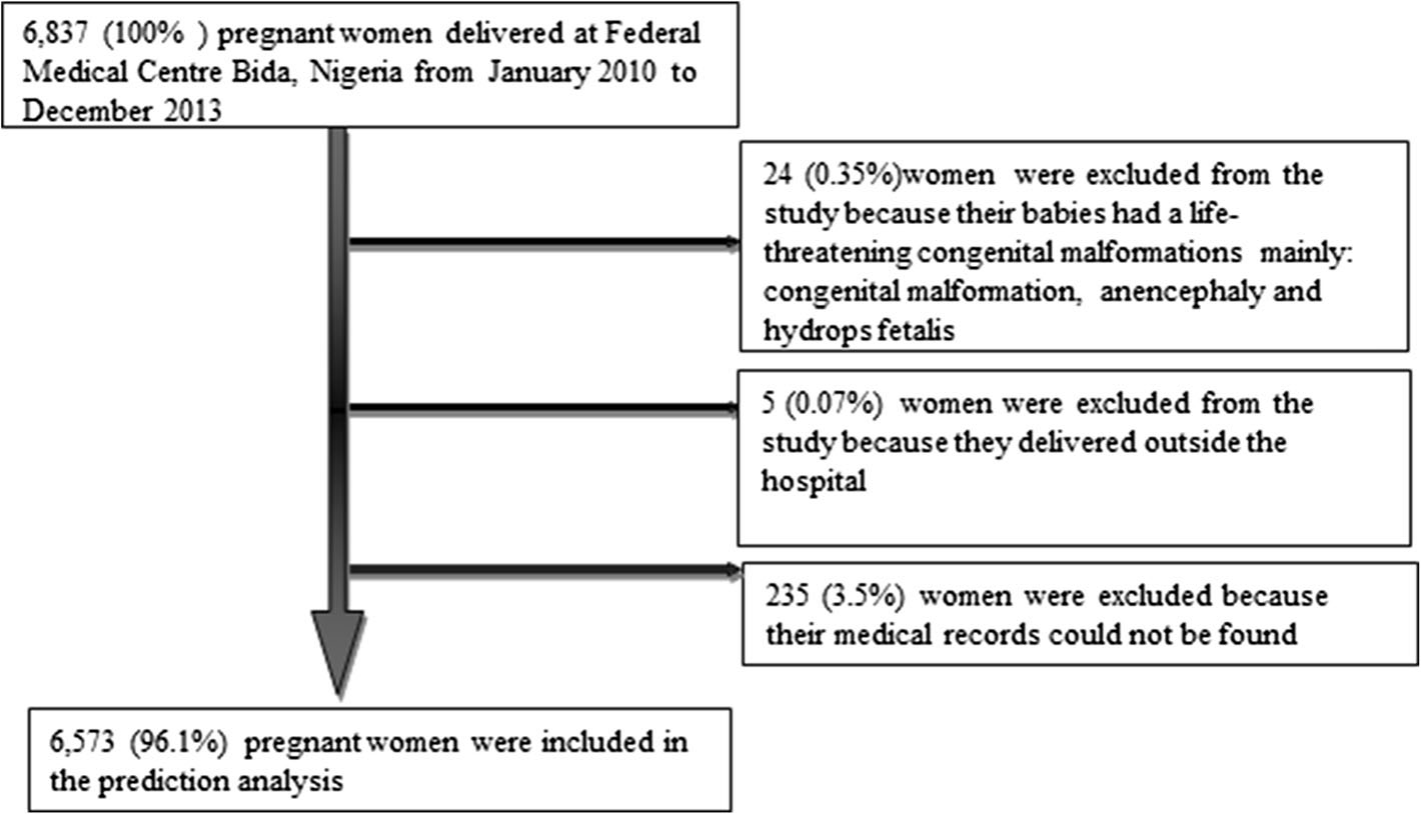
Follow up of study participants

Of the 6,808 pregnant women who were recorded to have given birth in the delivery register; 6,573 (96.5 %) of them were recruited into this study based on the inclusion criteria as shown in Fig. 1. A total of 6,956 newborns were delivered, 443 of them were lifeless at birth meaning that six in 100 newborns delivered at this center were lifeless at birth. Table 1 shows the descriptive characteristics of the study population and percentage of missing data in each characteristic of the patients. The median age of women who delivered at the center was 27 years with an average parity of two. About two-thirds of the women had at least primary education; half of them were unemployed. The Nupe ethnic group accounted for 72 % of the women and they dwelled mainly in the urban areas (89 %). Almost 90 % of the babies delivered were singleton fetuses in cephalic presentation and 51 % were male. The median birth weight was 3.1 kg and the mean gestational age at birth was 39 weeks. Average percentage of missing data per potential predictor was 11 %. About 23 % of the women have co-existing medical conditions, 30 % of them were nulliparous while one quarter of them have had fetal loss (Additional file 1: Table S1).

**Table 1:** General characteristics of the study population

Characteristics	All women	Live infant (6,513)	Stillbirth (443)	Missing data [%]
Maternal age (years)	27 (24; 30)	26 (24; 30)	27 (25; 30)	0.5
Maternal height (centimeters)	156(153; 160)	156 (152; 160)	156 (153; 161)	32.1
Maternal weight (Kg)	65 (57; 75)	66.0 (57; 75)	66.0 (54.5; 75.5)	30.3
Parity	2(0; 3)	1 (0; 3)	3 (0; 4)	1.6
Number of previous fetal loss	0 (0; 1)	0 (0; 1)	0 (0; 1)	1.8
Number of previous caesarean section	0 (0; 0)	0 (0; 0)	0 (0; 0)	9.0
Maternal comorbidity	0 (0; 0)	0 (0; 0)	0 (0; 0)	1.5
Birth weight (Kg)	3.1 (2.7; 3.4)	3.1 (2.8; 3.4)	2.8 (2.0; 3.2)	4.1
Gestational age at birth (days)	265 (137; 276)	266 (137; 276)	225 (140; 254)	30.8
Maternal education (Educated)	3,284 [63.8]	3,171 [96.6]	113 [3.4]	26.0
Maternal education (Not educated)	1,866 [36.2]	1,747 [96.6]	119 [6.4]	
Male infant	3,506 [51.4]	3,287 [93.6]	219 [6.3]	2.0
Female infant	3,310 [48.6]	3,113 [94.0]	197 [6.0]	
Bleeding in pregnancy (Yes)	341 [5.1]	220 [64.5]	121 [35.5]	3.0
Bleeding in pregnancy (No)	6,406 [94.9]	6,107 [95.3]	299 [4.8]	
Maternal occupation				16.2
Not employed	2,894 [49.6]	2,650 [91.6]	244 [8.4]	
Self-employed	1,969 [33.8]	1,884 [95.7]	85 [4.3]	
Private/public employee	968 [16.7]	930 [96.1]	38 [3.9]	
Ethnicity				7.7
Nupe	4,611 [71.9]	4,297 [93.2]	314 [6.8]	
Hausa / Fulani	246 [3.8]	220 [89.4]	26 [10.6]	
Yoruba	790 [12.3]	758 [95.9]	32 [4.1]	
Igbo	395 [6.2]	378 [95.7]	17 [4.3]	
Gwari	19 [0.3]	17 [89.5]	2 [10.5]	
Others	356 [5.6]	342 [96.1]	14 [3.9]	
Place of residence (Urban)	5,707 [89.1]	5,449 [95.5]	258 [4.5]	7.9
Place of residence (Rural)	700 [10.9]	552 [78.9]	148 [21.1]	
Multiple gestation				<0.01
Singleton	6,201 [89.2]	5,813 [93.7]	388 [6.3]	
Twins	719 [10.3]	665 [92.5]	54 [7.5]	
Triplets	35 [0.5]	34 [97.1]	1[2.86]	
Fetal presentation				<0.01
Cephalic	6,506 [93.7]	6,159 [94.7]	347 [5.3]	
Breech	334[4.8]	280 [83.8]	54 [16.2]	
Others	100[1.4]	62 [62.0]	38 [38.0]	

Median (interquartile range); number [percentage]

### Multivariable prediction model

The results of the multivariable prediction model for stillbirth (i.e. the basic model) are shown in Table 2. The final model comprised maternal comorbidity, place of residence, maternal occupation, parity, bleeding in pregnancy, and fetal presentation as independent predictors of stillbirth. For every morbid condition co-existing with pregnancy the likelihood of stillbirth increased. Being an unemployed, rural-dwelling woman with a positive history of bleeding in pregnancy increased risk of stillbirth. As parity increased risk of stillbirth increased. Pregnancies in cephalic presentation lowered the risk of stillbirth. Subsequently, the basic model was extended by the variable fetal growth rate and the results of the multivariable prediction model (i.e. the extended model) are shown in Table 3. All predictors in the extended model showed similar associations as observed in the basic model. For fetal growth rate, the likelihood of stillbirth decreased as growth rate increased.

**Table 2:** Multivariable prediction model for stillbirth (Basic model)

Predictors	*Unadjusted β* coef.	Standard error	*P*-value	Adjusted *β* coef.
Maternal comorbidity	0.71	0.097	<0.001	0.71
Place of residence (rural)	1.31	0.129	<0.001	1.30
Maternal occupation				
Self employed	−0.30	0.144	0.035	−0.30
Employee	−0.38	0.182	0.037	−0.38
Maternal parity	0.08	0.024	0.001	0.08
Bleeding (yes)	2.18	0.139	<0.001	2.16
Fetal presentation				
Breech	0.96	0.182	<0.001	0.96
Others	2.12	0.240	<0.001	2.06

Unadjusted *β* coef. denotes *β* coefficient before penalization; Adjusted *β* coef. denotes *β* coefficient after penalization

C-statistic before and after penalization 0.80 (95 % CI 0.78–0.83)

$$ \begin{array}{l}\mathrm{Risk}\ \mathrm{of}\ \mathrm{s}\mathrm{tillbirth} = 1\ /\ 1 + \exp \Big(\hbox{-} \left(\hbox{-} 3.6486 + 0.7077*\left(\mathrm{comorbidity}\right) + 1.3047*\left(\mathrm{rural}\right)\ \hbox{--} 0.3022*\left(\mathrm{self}\hbox{-} \mathrm{employed}\right)\hbox{-} \right.\\ {}0.3788*\left(\mathrm{employee}\right) + 0.0797*\left(\mathrm{parity}\right) + 2.1579*\left(\mathrm{bleeding}\ \mathrm{in}\ \mathrm{pregnancy}\right) + 0.9616*\left(\mathrm{breech}\ \mathrm{presentation}\right) + \\ {}\left.2.0588*\left(\mathrm{other}\ \mathrm{presentation}\mathrm{s}\right)\right)\end{array} $$

For example the risk of a para-7, unemployed, hypertensive, diabetic pregnant woman in compound presentation with a positive history of vaginal bleeding in pregnancy, dwelling in a rural area is

$$ \begin{array}{l}\mathrm{Risk}\ \mathrm{of}\ \mathrm{stillbirth} = 1\ /\ 1 + \exp \Big(\hbox{-} \left(\hbox{-} 3.6486 + 0.7077*(2) + 1.3047(1)\ \hbox{--}\ 0.3022(1)\ \hbox{--}\ 0.3788(0)+0.0797(7)+2.1579(1)\right.\\ {}\left.+\kern0.5em 0.9616(0)+2.0588(1)\right)\\ {}=1/1\kern0.5em +\kern0.5em  \exp \left(\hbox{-} 3.5439\right)\\ {}\mathrm{Risk}\kern0.5em \mathrm{of}\kern0.5em \mathrm{stillbirth}=0.97\end{array} $$

**Table 3:** Extended multivariable prediction model for stillbirth (Extended model)

Predictors	Unadjusted *β* coef.	Standard error	*P*-value	Adjusted *β* coef.
Maternal comorbidity	0.60	0.100	<0.001	0.60
Place of residence (rural)	1.27	0.129	<0.001	1.26
Maternal occupation				
Self employed	−0.27	0.143	0.07	−0.26
Employee	−0.33	0.183	0.07	−0.33
Maternal parity	0.10	0.024	<0.001	0.10
Bleeding (yes)	2.04	0.142	<0.001	2.01
Fetal presentation				
Breech	0.83	0.181	<0.001	0.83
Others	2.15	0.241	<0.001	2.07
Growth rate	−0.18	0.026	<0.001	−0.18

Unadjusted *β* coef. denotes *β* coefficient before penalization; Adjusted *β* coef. denotes *β* coefficient after penalization

C-statistic before and after penalization 0.82 (95 % CI 0.80–0.85)

$$ \begin{array}{l}\mathrm{Risk}\ \mathrm{of}\ \mathrm{s}\mathrm{tillbirth} = 1\ /\ 1 + \exp \left(\hbox{-} \right(\hbox{-} 1.7035 + 0.5965*\left(\mathrm{comorbidity}\right) + 1.2603*\left(\mathrm{rural}\right)\ \hbox{--}\ 0.2647*\left(\mathrm{self}\hbox{-} \mathrm{employed}\right)\ \hbox{--} \\ {}0.3265*\left(\mathrm{employee}\right) + 0.0959*\left(\mathrm{parity}\right) + 2.0149*\left(\mathrm{bleeding}\ \mathrm{in}\ \mathrm{pregnancy}\right) + 0.8342*\left(\mathrm{breech}\ \mathrm{presentation}\right) + \\ {}2.0677*\left(\mathrm{other}\ \mathrm{presentation}\mathrm{s}\right)\ \hbox{--}\ 0.1810*\left(\mathrm{fetal}\ \mathrm{growth}\ \mathrm{rate}\right)\end{array} $$

For example the risk of a para-five, unemployed, hypertensive pregnant woman in breech presentation with a positive history of vaginal bleeding in pregnancy, dwelling in a rural area and the estimated fetal weight by obstetric scan at 22 weeks was 650 g

$$ \begin{array}{l}\begin{array}{l}\mathrm{Risk}\ \mathrm{of}\ \mathrm{stillbirth} = 1\ /\ 1 + \exp \Big(\hbox{-} \left(\hbox{-} 1.7035 + 0.5965(1) + 1.2603(1)\ \hbox{--}\ 0.2647(1)\ \hbox{--}\ 0.3265(0) + 0.0959(5) + 2.0149(1)+\right.\\ {}\left.0.8342(1) + 2.0677(0)\ \hbox{--}\ 0.1810\left(650/22*7\right)\right)\ \end{array}\hfill \\ {}\begin{array}{l}\kern6.75em  = 1/1 + \exp \left(\hbox{-} 2.4532\right)\\ {}\mathrm{Risk}\ \mathrm{of}\ \mathrm{stillbirth} = 0.92\end{array}\hfill \end{array} $$

### Performance of the model

**Fig. 2:**
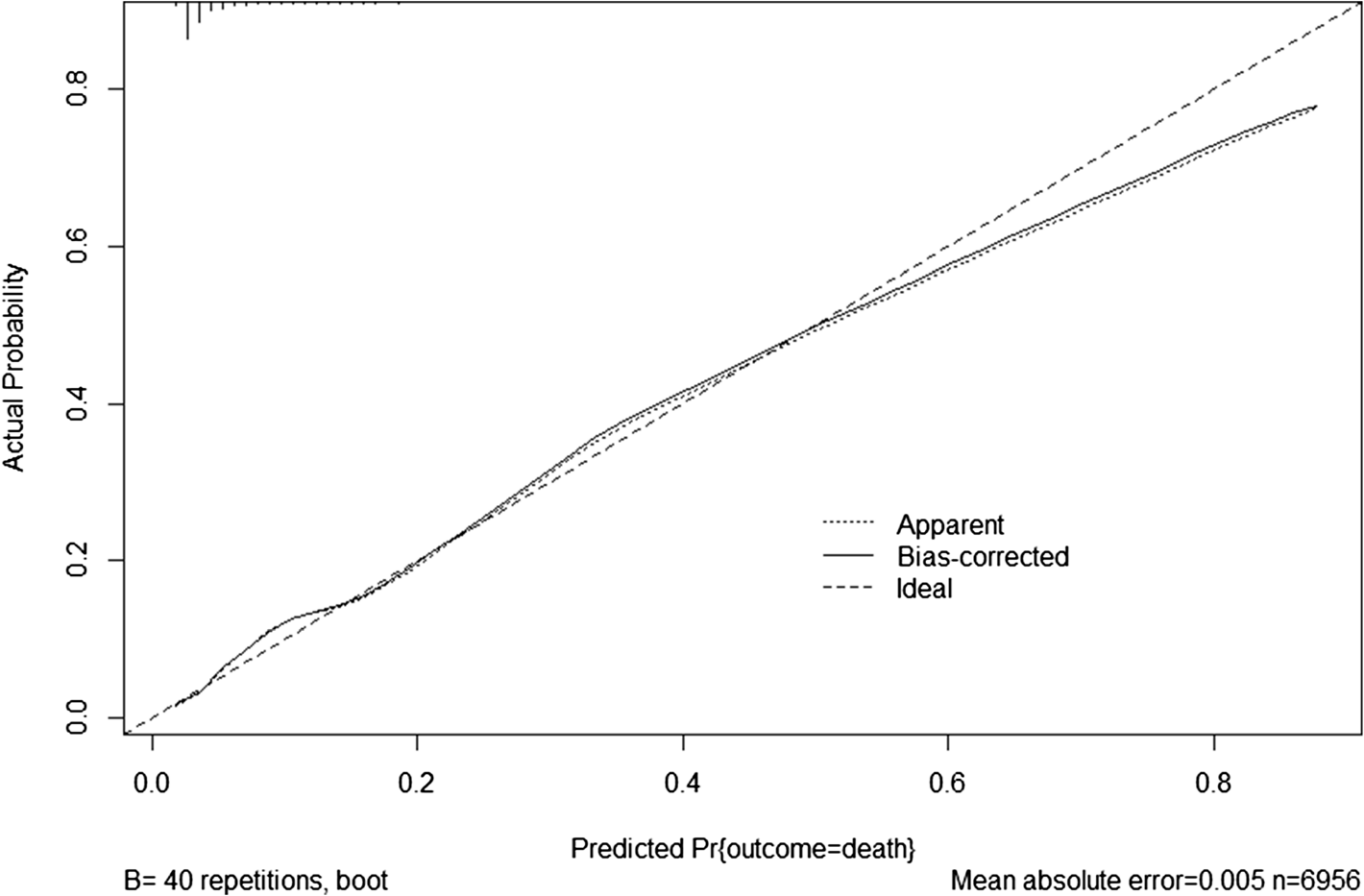
Calibration plot of the basic model

**Fig. 3:**
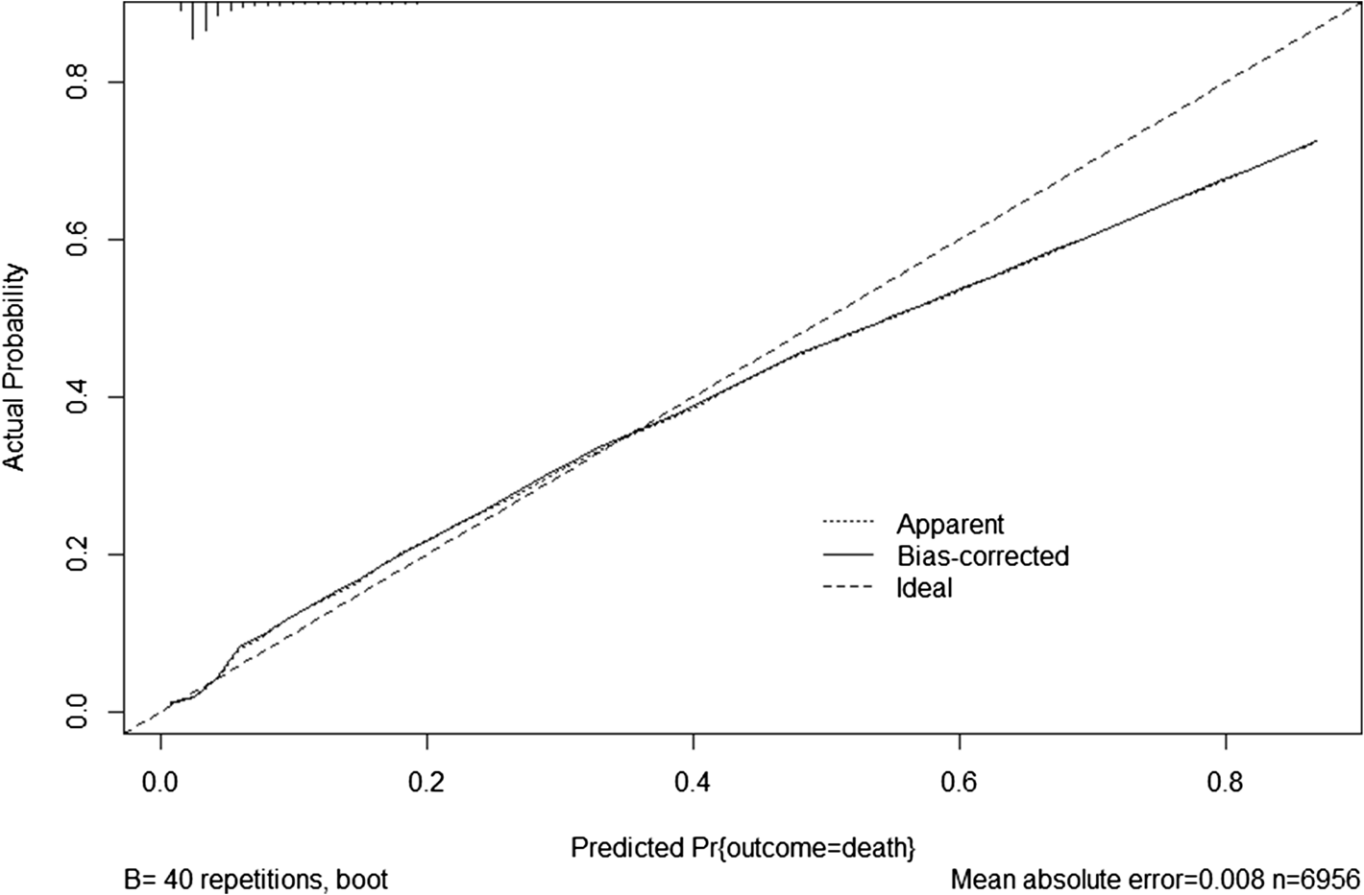
Calibration plot of the basic model

The discriminative performance of the final basic model was very good with a C-statistic of 0.80 (95 % CI 0.78–0.83). The extended model (i.e. with obstetric ultrasound variable growth rate added) showed a slightly improved discriminative performance of 0.82 (95 % CI 0.80–0.85). Calibration for both models was good (Figs. 2 and 3).

### Internal validation

Both models were penalized but the discriminative performance of both models remained unchanged while their calibration improved (Figs. 2 and 3).

## Discussion

In this study we developed an easy to use clinical prediction model to identify pregnancies at high risk of stillbirth for timely interventions. We also extended this basic model with the variable fetal growth rate (fetal weight divided by gestational age) to see whether this not routinely measured variable (obstetric ultrasound) would improve predictions. This study was reported based on the TRIPOD (Transparent reporting of a multivariable prediction model for individual prognosis or diagnosis) guidelines [39] and to the best of our knowledge, these are the first prediction models for stillbirth that can easily be applied in the second trimester of pregnancy in low-resource settings where 98 % of third-trimester stillbirths occur [4]. This study reaffirms stillbirth as an important public health issue; 6 in every 100 newborns delivered at the center were lifeless at birth, justifying the clinical relevance of an easy to use prediction model to detect high risk pregnancies at an early stage (i.e. the 2nd trimester). The basic prediction model comprised six easy-to-measure, readily available, inexpensive parameters, promoting its easy use during antenatal visits in low-resource settings. A previous model [27] included more predictors, but also used Pregnancy-Associated Plasma Protein-A and Reversed Flow of A-wave in Ductus Venosus that are not routinely measured in low-resource settings. Age restriction was not included in the eligibility criteria so as to broaden its application among pregnant women. A large cohort was used to develop the model to increase the power of the study and lower the possibility of overfitting. The predictive performance of the model in terms of discrimination and calibration was very good also after internal validation. As a secondary analysis we generated fetal growth rate using birth weight and gestational age at birth. This proxy predictor was included in the extended model (Table 3) instead of using ultrasound estimated fetal weight and gestational age, because up to 60 % of the women did not undergo obstetric ultrasound investigation during antenatal care due to various reasons. To acknowledge the importance of monitoring intrauterine growth restriction in stillbirth, fetal growth rate was included in the extended multivariable model. We preferred to generate fetal growth rate from birth weight and gestational weight at birth instead of using obstetric ultrasound information because based on our knowledge of these data some of the reasons why obstetric ultrasound was not done might be related to the outcome e.g. antenatal visit. Missing data was observed in some of our predictors and multiple imputation was applied to address it instead of performing a complete case analysis which may give biased results. Studies have shown repeatedly that multiple imputation reduces the possibility of bias in the estimates compared to complete case analysis [40–42]. It is important to emphasize that this prediction model has not undergone external validation, and this is planned to be done in a future study; but its predictive performance remained unchanged after internal validation. Experts have expressed the need to develop a prediction model for stillbirth because of its clinical importance [12]. It allows for early detection of pregnancies at high risk of stillbirth for timely allocation of targeted interventions and to benefit from closer monitoring throughout the pregnancy. Prioritization of care allocation is particularly relevant in low resource settings. Interventions to improve neonatal, intrauterine and maternal survival have been identified and integrated as a continuum of care because they are related [5, 6]; thus, it is expected that this prediction model may not only improve prevention of stillbirth but may also have a positive collateral effect on maternal and neonatal survival.

## Conclusion

We developed a simple but informative prediction model for early detection of pregnancies at high risk of stillbirth for timely intervention in low resource settings. It is important for future studies to conduct an external validation of this prediction model at all levels of care using prospectively collected data and include information on maternal HIV status.

## Additional file

Additional file 1: Table S1. Describes characteristics of the women. (PDF 117 kb)

## Abbreviations

AIC: Akaike information criterion
FBS: Fasting blood sugar
LMICs: Low- and middle-income countries
RBS: Random blood sugar
TRIPOD: Transparent reporting of a multivariable prediction model for individual prognosis or diagnosis
