# Supplementary material for: Predicting stillbirth in a low resource setting
Source: BMC Pregnancy Childbirth. 2016 Sep 20;16:274. doi: 10.1186/s12884-016-1061-2 (PMC5029011; doi:10.1186/s12884-016-1061-2)
Supplement: Additional file 1: Table S1. — Describes characteristics of the women. (PDF 117 kb) [file 12884_2016_1061_MOESM1_ESM.pdf]

Additional file 1: Table S1

Maternal characteristics		Number [%]
Number of previous fetal loss		
	0	5,090 [74.5]
	1	1,207 [17.7]
	2	393 [5.7]
	3 or more	143 [2.1]
Number of previous caesarean section		
	0	5,914 [93.5]
	1	340 [5.4]
	2	63 [1.0]
	3	10 [0.1]
Parity		
	0 (Nulliparous)	2,051 [30.0]
	1	1,339 [19.6]
	2	1,126 [16.5]
	3	864 [12.6]
	4	630 [9.2]
	5 or more	836 [12.1]
Number of maternal comorbidity		
	0	5,287 [77.2]
	1	1,475 [21.5]
	2	87 [1.3]
	3	2 [0]
